# Anaemia and associated factors among children aged 6–23 months in agrarian community of Bale zone: a cross-sectional study

**DOI:** 10.1017/jns.2022.63

**Published:** 2022-11-02

**Authors:** Mekonnen Tegegne, Kalkidan Hassen Abate, Tefera Belachew

**Affiliations:** Department of Nutrition and Dietetics, Faculty of Public Health, Jimma University, Jimma, Ethiopia

**Keywords:** Anaemia, Child, Cross-sectional, Haemoglobin, Infant, AOR, adjusted odds ratio, CI, confidence interval, COR, crude odds ratio, EDHS, Ethiopian Demographic and Health Survey, FANTA, Food and Nutritional Technical Assistance, GPS, global positioning system, HAZ, height-for-age *Z*-score, Hb, haemoglobin, HFIAS, Household Food Insecurity Access Scale, HHFSS, household food security status, IDA, iron deficiency anaemia, INNP, National Nutritional Program, IYCF, Infant and Young Child Feeding, OR, odds ratio, PCA, principal component analysis, WAZ, weight-for-age *Z*-score, WHO, World Health Organization, WHZ, weight-for-height *Z*-score

## Abstract

Anaemia remains among the most prevalent nutritional problems among children in developing countries. In Ethiopia, more than half of children <5 years of age are anaemic. In the early stages of life, it leads to poor cognitive performance, delay psychomotor development and decreases working capacity in later life. The present study aimed to assess the prevalence and associated factors of anaemia among children aged 6–23 months in the Bale zone. A community-based cross-sectional study was conducted from 1 to 30 June 2021. Multistage stratified sampling and simple random sampling techniques were employed to select 770 samples. An interviewer-administered questionnaire was used to collect data on socio-demographic, child health and feeding practices. Haemoglobin levels were estimated using a portable Hemosmart machine. Children with haemoglobin values below 11 g/dl were considered anaemic. Binary logistic regression analysis was performed to identify factors associated with anaemia. Statistical significance was set at *P* < 0⋅05. The prevalence of anaemia was 47⋅9 % (95 % CI (44⋅4, 51⋅5)). The multivariate analysis showed that child age (6–11 months) (AOR 1⋅47; 95 % CI (1⋅06, 2⋅03)), household food insecurity (AOR 1⋅44; 95 % CI (1⋅01, 2⋅04)), having diarrhoea and cough in the past 2 weeks (AOR 1⋅70; 95 % CI (1⋅18, 2⋅44)) and (AOR 1⋅97; 95 % CI (1⋅28, 3⋅04), respectively), not consuming the recommended dietary diversity (AOR 2⋅72; 95 % CI (1⋅96, 3⋅77)) and stunting (AOR 1⋅88; 95 % CI (1⋅31, 2⋅70)) were significantly associated with anaemia. Anaemia in children aged 6–23 months was a severe public health problem in the study area. Integrated nutritional interventions combined with iron fortification and supplementation is recommended.

## Introduction

Anaemia, a condition marked by low levels of haemoglobin (Hb) concentration in red blood cells (RBCs), affects approximately one-fourth of the world's population^(1,2)^. It is a public health problem in both developed and developing countries^(3)^. Although anaemia occurs at all stages of life, infants and young children are at elevated risk because of their rapid growth and development. In addition, their stored iron gets deficient during this period^(4)^. Globally, 43 % of children under the age of 5 are anaemic with a higher prevalence in Africa and Asia. In 2016, the estimated prevalence was approximately 55 % in South Asia and 60 % in Sub-Saharan Africa^(5)^.

The causes of anaemia in low- and middle-income countries are multifactorial. Even though, there may be many causes dietary iron deficiency is usually the major contributing factor. Other significant nutritional deficiencies (e.g. low intakes of folic acid and vitamin A, B12 and C) and infectious diseases (e.g. malaria and hookworm) may also contribute to anaemia^(6,7)^. In resource-limited settings, the World Health Organization (WHO) recommended haemoglobin concentration level to assess anaemia in under-five children with a cut-off point of less than 11⋅0 grams per decilitre (g/dl)^(8)^. Anaemia in childhood has an irreversible adverse effect on the health, growth and development of children^(9)^. A child with anaemia will have repeated episodes of infection and infection episodes are associated with risk of morbidity and mortality^(10)^. Children suffering from iron deficiency anaemia (IDA) have also slower cognitive development and poor school performance and work capacity in later years, which intern reduces the earning potential of individuals and hence damages national economic growth at large^(11,12)^.

The Ethiopian government has made different strategies to alleviate childhood malnutrition in all its forms. For example, the National Nutritional Program II (NNP-II) has planned to reduce anaemia in children under five to 24 % by 2020 through initiatives like identifying and treating anaemia cases, fortification of food and providing micronutrient supplements^(13)^. Despite all efforts, anaemia remains a serious challenge in Ethiopian children. The global burden of diseases study showed that anaemia in children was one of the most common causes of child death in Ethiopia, and continues to be a major public health problem^(14)^. In addition, the 2016 Ethiopia Demographic and Health Survey (EDHS) reported that the prevalence of anaemia among children 6–59 months of age was 57 %. The levels were higher in those aged 6–23 months, with 72 % of these children having anaemia. With a prevalence level higher than 40⋅0 %, the WHO considers anaemia in children as a severe public health problem. Most importantly because of differences in the geographical area and socio-economic characteristics, the magnitude of anaemia showed regional variation ranging from (83 %) in Somali region to (42 %) in Amhara region. Oromia region where this study was conducted is among the highest prevalence (66 %)^(15,16)^. Previous studies have reported that childhood anaemia is associated with a higher risk of continued breast-feeding, early initiation of complementary feeding, poor dietary diversity, having household food insecurity, drinking water from unsafe sources, not receiving anthelminthic drugs and stunting^(17–19)^.

Despite their increased vulnerability, little was known about the magnitude and associated factors of anaemia among infants and young children aged 6–23 months in the study area. Identifying the magnitude, distribution and risk factors of IDA in different contexts in countries like Ethiopia with different lifestyle and cultural practices is a crucial step in eradicating the consequences of childhood anaemia. Moreover, information on the magnitude and factors associated with childhood anaemia in different settings and age groups provide important inputs to design inclusive strategies to achieve sustainable development goal 3 ‘Ensure healthy lives and promote well-being for all at all ages’. Therefore, the present study intended to assess the magnitude and associated factors of anaemia among children 6–23 months in Bale Zone, South-East Ethiopia.

## Methods and materials

A community-based cross-sectional study was conducted from 1 to 30 June 2021 among children aged 6–23 months in the Bale zone, to determine the magnitude of anaemia and its associated factors. Bale zone is one of the twenty administered zones in Oromia regional state, located in the southeastern part of Ethiopia. The capital of Bale zone is Robe town which is located 430 km away from Addis Ababa, the national capital. According to the Bale zone health office, the zone has an estimated population of 269 950 in 2019 of which 208 653 are under five and 72 514 are under two children. There is 1 referral, 2 genera, 1 primary hospital, 54 health centres and 223 health posts in the Zone. Farming and livestock keeping are the largest source of livelihood and wheat barely teff and legumes are the major crops grown in the zone.

### Sample size, sampling technique and procedures

The present study is the baseline for a quasi-experimental study conducted to assess the effect of soaking complementary food flours on haemoglobin, nutrition and health status of children 6–23 months in an agrarian community of Bale Zone (Clinical trial.gov NCTO5254717). The sample size for the baseline survey was determined using single population proportion formula with the following assumption: confidence interval (CI) of 95 %, a margin of error of 5 %, design effect of 2, non-response rate of 10 % and the proportion of child anaemia (65⋅5 %), stunting (37 %), underweight (24 %), and wasting (10 %) taken from EDHS 2016 for Oromia region^(16)^. Since the sample size calculated for the variable stunting is the largest = 787, it was taken as the estimated sample size for this study.

Multistage stratified followed by a simple random sampling procedure was employed to reach the study subjects. In the first stage out of seven agrarian districts in the Bale zone, two districts namely Agarfa and Goba were selected randomly. Kebeles (the smallest administrative unit in Ethiopia) found in the selected districts were stratified as urban and rural. In the selected districts, a total of eight (six rural and two urban) kebeles were selected randomly. After the total number of households with children aged 6–23 months in each selected kebeles was obtained from the health extension workers registry, the total sample size was proportionally allocated for each selected kebeles. At the third stage, a list of identification numbers for each household with an eligible child (a child aged 6–23 months) in randomly selected kebeles was developed and study participants were selected using a computer-generated simple random sampling method.

### Data collection and measurements

A pretested, semi-structured and interviewer-administered questionnaires which were developed by reviewing different relevant literature^(9,16,18)^, and guidelines^(19,20)^, were used to collect data. Questionnaires were initially prepared in English and translated into each local language the participants spoke; (Afaan Oromo and Amharic) and retranslated back to English to maintain consistency. The questionnaire was composed of data on household socio-demographic and economic status, Household Food Security Status (HHFSS), maternal and child health, child feeding practices and household's source of drinking water and availability of latrine. Household wealth was assessed using questioner adapted from the Ethiopian demographic and health survey (EDHS 2016) and household food insecurity was determined by using the Household Food Insecurity Access Scale (HFIAS) developed by Food and Nutritional Technical Assistance (FANTA). The HFIAS tools have nine occurrence questions that represent an increasing level of severity of food insecurity (access) and nine ‘repetitiveness of occurrence’ questions that are asked as a follow-up for each occurrence question to determine how often the condition occurred during the last 4 weeks. The frequency of occurrence of the event was ranked as ‘rarely’ (1), ‘sometimes’ (2) and ‘often’ (3). Scores to the answer of each question were summed to create a household food security score, with a minimum score of ‘0’ and a maximum score of ‘27’. The higher the score, the more food insecurity the household experience, and the lower the score, the less food insecurity the household experiences. Households with an HFIAS score of 0–1 are categorised as food secure and 2 and above were considered as food insecure^(21)^.

Child meal frequency and dietary diversity scores were determined by using tools of WHO Infant and Young Child Feeding (IYCF) indicators with some modifications to fit the local context. This is based on the mother's recall of all food her child consumed and the number of times her child took solid, semi-solid, or soft food in the previous 24 h. Children's health status was assessed from the history of a symptom of cough, fever and diarrhoea in the last 2 weeks preceding the survey.

The anthropometric measurement (length and weight) which is used to determine a child nutritional status were taken using standard techniques. The length of the child was taken in the recumbent position using a wooden measuring board and recorded to the nearest 0⋅1 cm, while weight was taken using a weighing scale with minimum clothing and recorded to the nearest 10 g.

Haemoglobin levels were determined using portable Hemosmart Gold (England, Serial No.Ax006/04) machine within the home by trained data collectors. For Haemoglobin test, a sample of blood was obtained by pricking fingertip of older children and heel of smaller children and the pricked finger or heel was gently pressed to get a sample of a drop of blood on the microcuvette and then the microcuvette was inserted into the Hemosmart machine. To prevent contamination of the blood sample, the finger or heel was cleaned with an alcohol swab and the first drop of blood was wiped off with clean cotton and the next drop was collected into a disposable microcuvette. After adjusting for altitude, Haemoglobin level was recorded to the nearest 0⋅1 g/dl. The altitude of the area's was measured by using a portable GPS (global positioning system) (GPS 72H GARMIN Idn. 1T72400267-Taiwan). Equipment like lancet, microcuvette and gloves were used for each child and discarded properly after use.

Data were collected by eight BSc Nurses and supervised by two Health officers who are fluent in speaking both Afaan Oromo and Amharic languages.

### Data quality assurance

The questionnaire was pretested in 5 % of the total sample before actual data collection outside the selected districts and modification was made based on the findings. Three days training were given to data collectors and supervisors. The focus of the training was on the objective of the study, interview techniques, basic skills of haemoglobin and anthropometric measurements, and on calibrations of equipment. The training also covered measures to be taken to prevent the transmission of COVID-19 during the data collection process.

To maintain the accuracy of weight measurement, the weight scale was returned to 0 before every measurement and calibrated using 1 kg standard weight. While the length measuring board was checked with other meter taps on daily bases. For each measurement of length and weight, reading was taken twice, and in cases when there was a difference the average of the two was taken.

### Operational definitions

- *Anaemia among children 6–23 months:* A child is considered to be anaemic if the adjusted haemoglobin count is less than 11⋅0 grams per decilitre (g/dl) against the World Health Organization (WHO) reference range. Haemoglobin value of 10–10⋅9 g/dl, 7⋅0–9⋅9 g/dl and less than 7 g/dl were considered as mild, moderate and severe anaemia, respectively^(20)^.
- *Stunting:* A child is considered to be stunted when a child's length-for-age *Z*-scores was less than −2 Standard Deviation (sd) from the sex-specific reference population of the World Health Organization (WHO) Multicentre Growth Study^(22)^.
- *Wasting:* A child is considered to be wasted when the child's weight-for-length *Z*-scores were less than −2 Standard Deviation (sd) from the sex-specific reference population of the World Health Organization (WHO) Multicentre Growth Study^(22)^.
- *Underweight:* A child is considered to be underweight when a child's weight-for-age Z-scores were less than −2 Standard Deviation (sd) from the sex-specific reference population of the World Health Organization (WHO) Multicentre Growth Study, was defined as underweight^(22)^.
- *Minimum meal frequency:* Breast-fed and non-breast-fed children aged 6–23 months who received solid, semi-solid or soft foods (but also including milk feeds for non-breast-fed children) with the minimum number of times or more. For breast-feeding, children minimum is defined as two times for infants 6–8 months and three times for children 9–23 months. For non-breast-feed, children minimum is defined as four times for children 6–23 months. ‘Meals’ include both meals and snacks (other than trivial amounts), and frequency is based on caregiver reports^(23)^.
- *Minimum dietary diversity:* Consumption of four or more food groups from the WHO recommended seven food groups within 24 h day or night before the survey. The seven foods groups used for tabulation of this indicator are: grains, roots and tubers; legumes and nuts; dairy products (milk, yogurt, cheese); flesh foods (meat, fish, poultry and liver/organ meats); eggs; vitamin-A-rich fruits and vegetables and other fruits and vegetables^(23)^.
- *Diarrhoea:* The passage of three or more loose or watery stools over 24 h period or more frequently than normal for a child in the last 2 weeks^(24)^.
- *Fever:* Mothers’ perception of increased body temperature per day in the last 2 weeks^(24)^.
- *Cough:* Mother's perception of cough in the last 2 weeks^(25)^.

### Data management and analysis

Data were checked for completeness, coded and entered into Epidata version 3.1 and exported to SPSS version 23.0 for analysis. The household wealth index was assessed based on household asset data using principal component analysis (PCA). Kaiser–Meyer–Olikin (KMO) test and Bartlett's Test of Sphericity (BTS) were done to determine sampling adequacy for PCA. To check the pattern of relationships between variables and components of communality was determined for every item, and items with communality less than 50 % were removed from the analysis. Components with Eigenvalues greater than 1, total variance explained more than 60 % and items loaded of at least 0⋅40 were retained to construct factor scores. Finally, factor scores computed by the PCA were summed and ranked as tertile (low, medium and high)^(26)^.

The WHO Anthro version 3.2.2 software was used to convert the anthropometric measures; weight, length and age values to *Z*-scores of the indices, and the WHO nutrition indices were used to classify the nutritional status as stunting, wasting and underweight^(27)^.

Descriptive statistics were used to summarise the characteristics of the study subjects. Bivariate and multivariate logistic regression analysis was carried out to assess any association between each independent variable and the dependent variable. Independent variables found to have *P*-value less than 0⋅05 at bivariate logistic regression were included in multivariable logistic regression for controlling the possible effect of confounders. Those variables with *P* < 0⋅05 in multivariable logistic regression analysis were considered to have statistical significance. The characteristics of association were determined based on odds ratio (OR) with a 95 % CI. The goodness of model fit was tested using Hosmer–Lemeshow test at *P* > 0⋅05.

### Ethical clearance

This study was conducted according to the guidelines laid down in the declaration of Helsinki and all procedures involving study subjects were approved by the Institutional Review Board of Jimma University (Ref. No. JHRPG1/776/20). Permission letters were also secured from the Bale zone health bureau and the respective district health offices. Informed written consent was obtained from study subjects after a brief explanation of the risks and benefits of participating in the study. For the issue of confidentiality, a unique identification number was given to subjects. Children aged 6–23 months who appear with severe acute malnutrition were referred to a health facility for treatment. Maximum precautions per WHO guidelines for the prevention of COVID-19 transmission were taken throughout the data collection period. All data collectors and supervisors wore face masks throughout the data collection period. Studied mothers who appeared without face masks were provided face masks. Data collectors cleaned their hands and equipment with sanitizer (60 % alcohol) after every contact and each procedure.

## Results

### Socio-economic and demographic-related characteristics

A total of 770 respondents was included in the final analysis, giving a response rate of 97⋅8 %. The mean age (±sd) of children and mothers were 11⋅58 (±2⋅750) months and 25⋅57 (±4⋅863) years, respectively. One hundred and seventy-four (22⋅6 %) of mothers and thirty-seven (4⋅8 %) of fathers were unable to read and write. Of the surveyed households, 287 (37⋅3 %) earned less than one thousand Birr per month, 256 (33⋅2 %) were ranked at a low wealth index level and 307 (39⋅8 %) were food insecure (Table 1).

**Table 1.** Socio-demographic and economic status of respondents in agrarian community of Bale zone, South East, Ethiopia, 2021 (*n* 707)

Characteristics	Category	Frequency	Percent
Child age	6–11	355	46⋅1
	12–23	415	53⋅9
Child sex	Male	389	50⋅5
	Female	381	49⋅5
Age of mothers	15–24	370	48⋅1
	25–34	367	47⋅7
	35 and above	33	4⋅3
Religion	Muslim	574	74⋅5
	Orthodox	174	22⋅6
	Protestant	19	2⋅5
	Catholic	3	0⋅4
Marital status	Married	689	89⋅5
	Unmarried	35	4⋅5
	Divorced	39	5⋅1
	Widowed	7	0⋅7
Educational status of mothers	Unable to read and write	174	22⋅6
	Read and write	257	33⋅4
	Primary school	221	28⋅7
	Secondary school	87	11⋅3
	College and above	31	4⋅0
Occupation of mothers	Housewife	567	73⋅6
	Governmental employee	70	9⋅1
	Daily labourer	54	7⋅0
	Merchant	79	10⋅3
Educational status of father (689)	Unable to read and write	37	4⋅8
	Read and write	222	28⋅8
	Primary school	218	28⋅3
	Secondary school	151	19⋅6
	College and above	61	7⋅9
Occupation of father (689)	Governmental employee	80	10⋅4
	Farmer	478	62⋅1
	Daily labourer	55	7⋅1
	Merchant	76	9⋅9
Number of children <5	1	307	39⋅9
	≥2	463	60⋅1
Birth order of the index child	1st child	242	31⋅4
	2nd child	314	40⋅8
	3rd child	97	12⋅6
	4th and above	117	15⋅2
Family size	0–4	361	46⋅9
	5–6	294	38⋅2
	Above 6	115	14⋅9
Place of residence	Urban	174	22⋅6
	Rural	596	77⋅4
Head of the HH	Mother	82	10⋅6
	Husband	688	89⋅4
Average monthly income of HH	≤1000 ETB	287	37⋅3
	1001–2000 ETB	309	40⋅1
	≥2000 ETB	174	22⋅6
Wealth category	Low	256	33⋅2
	Middle	236	30⋅6
	High	278	36⋅1
Food security	Yes	465	60⋅2
	No	307	39⋅8

HH, household; ETB, Ethiopian Birr.

### Maternal and child health-related characteristics

The majority 732 (95⋅1 %) of the mothers had received at least one antenatal care service (ANC), of which only 202 (26⋅1 %) have four or more ANC contacts during their last pregnancy. More than half 403 (52⋅3 %) of the mothers were taking iron folate supplementation, and the majority 653 (84⋅8 %) gave birth to their last child at the health facility. More than half 414 (53⋅8 %) of the mothers received postnatal service at least once during their last delivery. Nearly half 422 (54⋅8 %) of the children received vitamin A supplements in the previous 6 months and nearly one-third 227 (29⋅5 %) attended growth monitoring and promotion services. Four hundred and twenty-eight (55⋅6 %) of the studied children had a history of illness 2 weeks before this study. Diarrhoea was experienced by 208 (27⋅2 %), fever was experienced by 135 (17⋅5 %) and Cough was experienced by 126 (16⋅4 %) of the children 2 weeks before the survey (Table 2).

**Table 2.** Maternal and child health-related characteristics of respondents in agrarian community of Bale zone, South East, Ethiopia, 2021(*n* 707)

Characteristics	Category	Frequency	Percent
Attended ANC	Yes	732	95⋅1
	No	38	4⋅9
Number of ANC (*n* 732)	1–3	530	68⋅8
	4 and above	202	26⋅1
Counseling on IYCF (*n* 732)	Yes	464	60⋅3
	No	268	34⋅8
IFA	Yes	403	52⋅3
	No	367	47⋅7
Place of delivery	Health facility	653	84⋅8
	Home	117	15⋅2
PNC	Yes	414	53⋅8
	No	356	46⋅2
Number of PNC (*n* 414)	1	220	28⋅6
	Above 1	194	25⋅2
IYCF practice information during PNC services (*n* 414)	Yes	233	56⋅3
	No	181	43⋅7
Immunization status of children	Complete	260	33⋅8
	Not complete	510	66⋅2
Child deworming in the past 6 months	Yes	415	53⋅9
	No	355	46⋅1
Child vitamin A supplementation in the past 6 months	Yes	422	54⋅8
	No	348	45⋅2
Child growth monitoring and promotion service utilisation	Yes	227	29⋅5
	No	543	70⋅5
Child history of sickness 2 weeks before the survey	Yes	428	55⋅6
	No	342	44⋅4
Child history of diarrhoeal morbidity in the past 2 weeks	Yes	208	27⋅2
	No	562	73
Child history of fever in the past 2 weeks	Yes	135	17⋅5
	No	635	82⋅5
Child history of cough in the past 2 weeks	Yes	126	16⋅4
	No	644	83⋅6

ANC, antenatal care; IYCF, Infant and Young Child Feeding; IFA, iron folic acid; PNC, postnatal care.

### Child feeding practice

Six hundred and seventy-nine (88⋅2 %) of mothers fed colostrum to their children within the first 5 days after delivery and 679 (88⋅2 %) were reported giving pre-lacteal feeding. The majority 654 (84⋅9 %) of the mother were practicing exclusively breast-feeding for the first 6 months. Seven hundred and forty (96⋅1 %) children were introduced complementary feeding at age of 6 months. Nearly two-thirds 555 (72⋅1 %) of the children received the recommended minimum meal frequency and more than one-third 292 (37⋅9 %) of them received minimum dietary diversity. Regarding nutritional status, about 112 (14⋅5 %) of the children were wasted, 259 (33⋅6 %) were stunted and 87 (11⋅3 %) were underweight (Table 3).

**Table 3.** Child feeding practice-related characteristics of respondents in agrarian community of Bale zone, South East, Ethiopia, 2021

Characteristics	Category	Frequency	Percent
Colostrum feeding	Yes	679	88⋅2
	No	91	11⋅8
Pre-lacteal feeding	Yes	81	10⋅5
	No	689	89⋅5
Currently BF	Yes	749	97⋅3
	No	21	2⋅7
Exclusive breast-feeding practice	Yes	654	84⋅9
	No	116	15⋅1
Introduction of complementary feeding at 6 months	Yes	741	96⋅2
	No	29	3⋅8
Meet minimum meal frequency	Yes	555	72⋅1
	No	215	27⋅9
Meet minimum dietary diversity	Yes	292	37⋅9
	No	478	62⋅1
Wasted	Yes	112	14⋅5
	No	658	85⋅3
Stunted	Yes	259	33⋅6
	No	511	66⋅4
Underweight	Yes	94	12⋅2
	No	676	87⋅8

BF, Breast-feeding.

### Water source sanitation and hygienic

Six hundred and ninety-three (90 %) of the households were used pipe water as their source for drinking water. The majority of the households 697 (90⋅1) owned latrine/toilet in their house (Table 4).

**Table 4.** Water source sanitation and hygienic-related characteristics of respondents in agrarian community of Bale zone, South East, Ethiopia, 2021

Characteristics	Category	Frequency	Percent
	Piped inside compound	197	25⋅6
	Public tap	496	64⋅4
	Protected well/spring	11	1⋅4
	Unprotected dug/spring	66	8⋅6
Time spent to fetch water	<30 min	662	86⋅0
	>30 min	108	14⋅0
Latrine *n* 694	No latrine	76	9⋅9
	Have latrine	694	90⋅1
Type of latrine (*n* 694)	Pit	270	35⋅1
	Latrine with shade	404	52⋅5
	VIP	12	1⋅6
	Flush to sewage/septic tank	8	1⋅0

VIP, ventilated improved pit latrine.

### Prevalence of anaemia

**Fig. 1.**
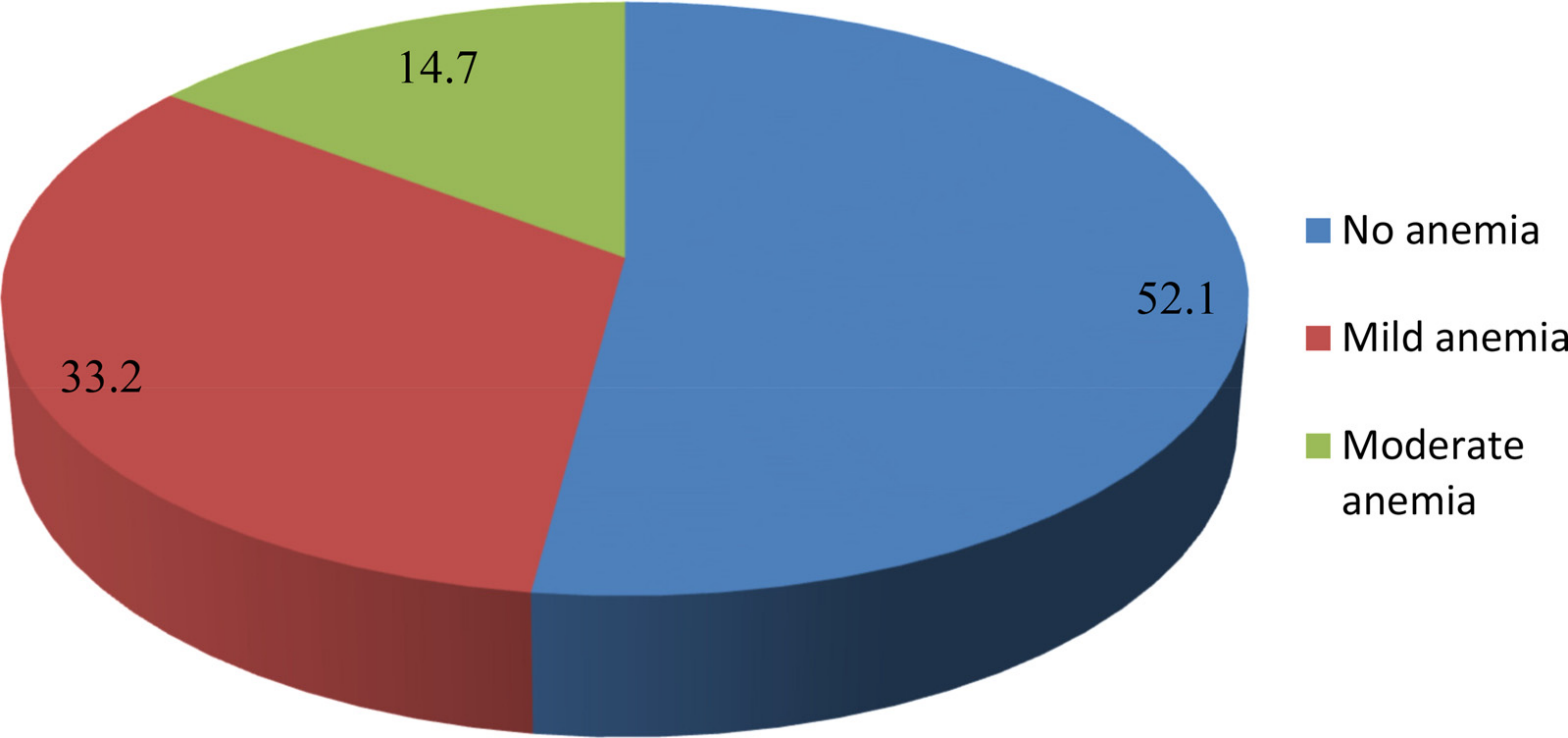
Prevalence of anaemia among children age 6–23 months in agrarian community of Bale zone, South East, Ethiopia, 2021.

The mean ± sd haemoglobin concentration was 11⋅23 ± 1⋅26 g/dl with a range of 7⋅8–15⋅6 g/dl. The overall prevalence of anaemia among children aged 6–23 months was **47⋅9 % (95 % CI 44⋅4, 51⋅5)**. Among anaemic children, 113 (14⋅7 %) had moderate anaemia, 256 (33⋅2 %) had mild anaemia and no child had severe anaemia. Anaemia was highest among children aged 6–11 months (52⋅1 %) than children aged 12–23 (44⋅3 %) (Fig. 1).

### Factors associated with anaemia

In Bivariable logistic regression analysis, child's age, maternal age, maternal educational status, household monthly income, household source of drinking water, availability of latrine, HHFSS, maternal iron folic acid (IFA) intake, child's growth promotion service utilisation, child's history of cough and diarrhoea in the past 2 weeks, child minimum dietary diversity practice, stunting and underweight were factors associated with anaemia at a *P*-value of less than 0⋅5. Subsequently, these variables were fitted to multivariate logistic regression model, and it was observed that child age, household food insecurity, child's history of cough and diarrhoea morbidity, poor dietary diversity practice and stunting were significantly associated with anaemia at a *P*-value of 0⋅05. According to the multivariable logistic regression analysis, the odds of anaemia among children aged 6–11 months were 1⋅47 times more likely to compare with those aged 12–23 months (AOR 1⋅47; 95 % CI (1⋅06, 2⋅03)). Similarly, the odds of anaemia children among food insecure household were 1⋅44 times more likely to compare with those from food secure households (AOR 1⋅44 (1⋅28; 95 % CI (1⋅01, 2⋅04))). Having cough morbidity in the past 2 weeks before the survey (AOR 1⋅97; 95 % CI (1⋅28, 3⋅04)) and diarrhoea morbidity in the past 2 weeks before the survey (AOR 1⋅70; 95 % CI (1⋅18, 2⋅44)) were also associated with increased odds of anaemia. Higher odds of anaemia were observed among children who did not receive minimum dietary diversity (AOR 2⋅72; 95 % CI (1⋅96, 3⋅77)). Relative to not stunted children, increased odds of anaemia were observed among stunted children (AOR 1⋅88; 95 % CI (1⋅31, 2⋅70)) (Table 5).

**Table 5.** Factors associated with anaemia among children aged 6–23 months, agrarian community of Bale zone, South East, Ethiopia, 2021

Characteristics	Category	Anaemia		COR	AOR
		Yes	No		
Child age	6–11	185	170	1⋅37(1⋅02–1⋅81)^a^	1⋅47(1⋅06–2⋅03)^a^
	12–23	184	231	1	1
Age of mothers	15–24	179	191	0⋅46(0⋅22–0⋅99)**a**	0⋅54(0⋅23–1⋅27)
	25–34	168	199	0⋅42(0⋅19–0⋅89)^a^	0⋅43(0⋅19–0⋅99)
	35 and above	22	11	1	1
Educational status of mothers	Unable to read and write	95	79	3⋅46(1⋅47–8⋅15**)^a^**	1⋅73(0⋅61–4⋅84)
	Read and write	127	130	2⋅80(1⋅21–6⋅51)^a^	1⋅70(0⋅68–4⋅56)
	Primary school	101	120	2⋅42(1⋅03–5⋅64)^a^	1⋅54(0⋅58–4⋅07)
	Secondary school	38	49	2⋅23(0⋅89–5⋅53)	1⋅36(0⋅50–3⋅70)
	College and above	8	23	1	1
Average monthly income of HH	≤1000 ETB	147	140	**1⋅67(1⋅13–2⋅44)**^a^	0⋅95(0⋅58–1⋅54)
	1001–2000 ETB	155	154	1⋅61(1⋅10–2⋅36)^a^	1⋅07(0⋅69–1⋅68)
	≥2000 ETB	67	107	1	1
Source of drinking water	Piped inside compound	86	111	0⋅53(0⋅30–0⋅94)^a^	0⋅72(0⋅36–1⋅45)
	Public tap	236	260	0⋅62(0⋅37–1⋅05)	0⋅83(0⋅45–1⋅52)
	Protected well/spring	8	3	1⋅84(0⋅44–7⋅59)	3⋅25(0⋅72–14⋅72)
	Unprotected dug/spring	39	27	1	1
Availability of latrine	No latrine	45	31	1⋅65(1⋅02–2⋅68)^a^	1⋅24(0⋅71–2⋅16)
	Have latrine	324	370	1	1
Household food security	No	166	132	1⋅54(1⋅15–2⋅06)^a^	1⋅44(1⋅01–2⋅04)^a^
	Yes	203	269	1	
IFA	No	197	170	1⋅55(1⋅17–2⋅06)^a^	1⋅29(0⋅93–1⋅78)
	Yes	172	231	1	
Growth monitoring service utilisation	Yes	96	131	1⋅38(1⋅00–1⋅89)^a^	1⋅37(0⋅97–1⋅96)
	No	273	270	1	1
Cough	Yes	75	51	1⋅75(1⋅18–2⋅58)^a^	2⋅00(1⋅29–3⋅08)^a^
	No	294	350	1	1
Diarrhoea	Yes	117	96	1⋅47(1⋅07–2⋅02)^a^	1⋅70(1⋅18–2⋅44)^a^
	No	252	305	1	1
Meet minimum dietary diversity	Poor	274	197	2⋅98(2⋅20–4⋅05)^a^	2⋅74(1⋅97–3⋅80)^a^
	Good	95	204	1	
Stunted	Yes	158	101	2⋅22(1⋅63–3⋅01)^a^	1⋅81(1⋅26–2⋅60)^a^
	No	211	300	1	
Underweight	Yes	54	40	1⋅54(1⋅00–2⋅39)^a^	1⋅04(0⋅62–1⋅73)
	No	315	361		

ETB, Ethiopian Birr; HH, household; OR, odds ratio; CI, confidence interval, 1 = reference.

a Statistically significant association at *P* < 0⋅05.

## Discussion

The study revealed that the prevalence of anaemia among children aged 6–23 was 47⋅9 %. According to the WHO classification, anaemia becomes a severe public health problem when the magnitude is above 40 % in certain population groups^(28)^. Thus, the magnitude of anaemia in children 6–23 months in the study area was classified as a severe public health problem. A similar magnitude was reported in Deber Berhan town, North Shewa Ethiopia (47⋅5 %)^(29)^, Damot Sore district, South Ethiopia (52⋅6 %)^(30)^, Brazil (51 %)^(31)^ and Romania (46 %)^(32)^. However, the finding was higher than the study conducted in Huaihua china (29⋅73 %)^(33)^ and Northern Angola (44⋅4 %)^(34)^. The possible explanation for the variations in magnitude could be due to geographical and seasonal variations of risk factors and differences in the socio-economic status of the population. On the other hand, the prevalence of anaemia in children in this study is lower than the prevalence of the EDHS 2016 regional report for the Oromia region which was 66 %^(16)^. The discrepancies may be due to variations in the data collection period and the change in access and utilisation of health services by subjects’ overtime. Moreover, the present study was conducted among agrarian communities while the EDHS data include both agrarian and pastoralist communities. People living in pastoralist communities commonly feed their children camel, cattle and goat milk which are known for inhibiting iron absorption^(35)^. The result of the present study is also lower than studies conducted in agro-ecological zones of rural Ethiopia (53⋅7 %)^(36)^, Wag-Himra zone in North Ethiopia (66⋅6 %)^(37)^, Egypt (66 %)^(38)^ and rural Cameroon (66⋅7 %)^(39)^. The possible reason for this discrepancy could be due to differences in socio-economic status, place of residence and feeding practices. In addition, the lower occurrence of anaemia in the study area might be related to the lower prevalence of malaria.

Child age, household food security, having cough and diarrhoea morbidity 2 weeks before the study, dietary diversity practice and stunting were variables that have shown a significant association with childhood anaemia.

According to the present study, children aged 6–11 months had significantly higher odds of being anaemic as compared with children aged 12–23 months. The present study's finding is in line with other studies conducted in Somali region, Eastern Ethiopia^(40)^. Kilte Awulaelo Woreda, Northern Ethiopia^(41)^, Hohoe Municipality, Ghana^(42)^ and Bangladesh^(43)^. This could be due to the inadequate iron supply by breast milk despite a high iron requirement to support the rapid body growth and development at this age^(44)^. Moreover, the low iron containing plant-based monotonous foods feed to children of developing countries during the early stage of complementary feeding, may put them at higher risk of developing anaemia^(45)^.

Household food insecurity, a condition in which household members lack access to adequate food because of limited resources, is another factor that showed significant association with anaemia. This finding is supported by similar studies conducted in Damot Sore District, Wolaita Zone, South Ethiopia^(17)^, Wag-Himra zone North Ethiopia^(37)^ and Indonesia^(46)^. This could be because children from food insecure households are less likely to get essential nutrients including iron and important micronutrients such as vitamin A and C, which are very important for the bioavailability of iron. In addition, household food insecurity has been associated with caregiver depression and anxiety, which interferes with caregiver practice and adversely impacts children's well-being^(47)^.

Having diarrhoea and cough 2 weeks before the study were also significantly associated with anaemia. The finding is supported by studies done in the Wag-Himra zone, North Ethiopia^(37)^ Deber Berhan town, North Shewa Ethiopia^(29)^ and Burmac^(48)^. Several mechanisms can explain the higher odds of anaemia among children with infectious diseases. Infectious diseases can decrease intake and absorption of nutrients, cause intestinal mucosa injury and induce autoimmune reactions leading to anaemia.

Another factor that showed association with anaemia was dietary diversity practice. This is consistence with studies conducted in Damot Sore district, Southern Ethiopia^(17)^ and China^(47)^.

This could be because the more diversified a child's diet is, the larger the variety of nutrients he/she receives which enhances his/her health and nutrition. Increased dietary diversity is also associated with a higher likelihood of meeting children's recommended nutrient intake levels that may include important nutrients such as iron and other vitamins^(49)^. On the other hand, the negative association between anaemia and dietary diversity practice was observed in other studies^(48)^.

Stunted children were more likely to be anaemic compared with children who were not stunted. This is in agreement with a study conducted in Damot Sore district, Southern Ethiopia^(17)^, Dilla Town, Southern Ethiopia^(50)^, two agro-ecological zones of rural Ethiopia^(36)^ and Angola^(51)^. This could be because stunting is a consequence of malnutrition and it is a significant risk factor for anaemia. In addition, deficiencies of other micronutrients and stunting may synergistically increase the risk for anaemia.

The study has the following limitations. First, because of the cross-sectional nature of the design, a causal relationship cannot be established. Second, because the study only used haemoglobin values to determine anaemia status of children, a specific type of anaemia could not be determined. Third, recall and social desirability bias may also affect the IYCF and household food insecurity questionnaires.

## Conclusion

Anaemia in children aged 6–23 months was a severe public health problem in the study area. Being in the age group of 6–11, being from a food insecure household, having cough and diarrhoea morbidity, poor dietary diversity practice and stunting was significantly associated with child anaemia. The most critical period in human life for IDA to develop is 6–23 months of age because the iron requirement reaches the highest during this period, i.e. almost ten times higher by body weight than adults. According to the WHO, in the absence of special intervention such as fortification and supplementation, the bioavailability of iron is often poor, especially in developing countries where child diet is predominately monotonous plant source. Therefore, we recommend the concerned bodies to plan integrated nutritional intervention strategies combined with iron fortification and supplementation for tackling anaemia in this critical stage of life.
