# Anemia and associated factors among children aged 6-23 months in agrarian Community of Bale zone, a cross-sectional study – ADDENDUM

**DOI:** 10.1017/jns.2024.74

**Published:** 2024-11-18

**Authors:** Mekonnen Tegegne, Kalkidan Hassen Abate, Tefera Belachew

**Affiliations:** Department of Nutrition and Dietetics, Faculty of Public Health, Jimma University, Jimma, Ethiopia

The authors regret that the funding information was not included on the original publication of this article.

This should read:

Jimma University funded this research. The funders had no role in the study design, data collection, analysis, or manuscript production.
